# Arginine as host directed therapy in tuberculosis: insights from modulating arginine metabolism by supplementation and arginase inhibition

**DOI:** 10.1186/s44280-025-00070-6

**Published:** 2025-03-21

**Authors:** Qingkui Jiang, Ranjeet Kumar, Yi Zhao, Selvakumar Subbian, Lanbo Shi

**Affiliations:** 1 Public Health Research Institute, New Jersey Medical School, Rutgers Biomedical and Health Sciences, Rutgers, The State University of New Jersey, Newark, NJ 79103 USA; 2 Guangdong Provincial Key Laboratory of Medical Molecular Diagnostics, The First Dongguan Affiliated Hospital, Guangdong Medical University, Dongguan, Guangdong 523713 China; 3 Microbiology and Immunology Department, Guangdong Medical University, Dongguan, Guangdong 523808 China

**Keywords:** Arginase, Arginine metabolsim, Mitophagy, Cytokine network, Mycobacterium tuberculosis, Host directed therapy

## Abstract

Tuberculosis (TB), caused by *Mycobacterium tuberculosis* (*Mtb*), remains a global health challenge. Arginine metabolism is central to immune responses, regulating nitric oxide (NO) production via inducible NO synthase (Nos2) and competing pathways mediated by arginases (Arg1 and Arg2). This study examines the impact of arginine supplementation and arginase inhibition during the acute phase of *Mtb* infection in mouse lungs, focusing on immune function, lung pathology, and mitochondrial function. Arginine supplementation enhanced Nos2 expression, promoted mitophagy, and supported angiogenesis and/or tissue repair by upregulating *Vegfa*. These mechanisms synergized to balance pro-inflammatory responses with tissue repair, improving immune defense while mitigating lung damage. In contrast, arginase inhibition disrupted *Vegfa*-mediated immune homeostasis, and impaired mitophagy, leading to exacerbated lung pathology. These findings underscore the complementary roles of Nos2 and arginase-mediated pathways in maintaining immune equilibrium during *Mtb* infection. Our results highlight arginine supplementation as a promising host-directed therapy for TB, capable of enhancing protective immunity and facilitating tissue repair. Conversely, caution is warranted for strategies targeting arginase due to potential adverse effects on inflammation resolution and mitochondrial quality control. Future studies should explore the long-term efficacy of arginine-based therapies and their integration with existing antibiotic regimens for optimal TB management.

**Supplementary Information:**

The online version contains supplementary material available at 10.1186/s44280-025-00070-6.

## Introduction

Tuberculosis (TB), a chronic inflammatory disease caused by *Mycobacterium tuberculosis* (*Mtb*), has reemerged as the leading cause of death from a single infectious agent following the COVID-19 pandemic [1]. Effective immune defense against *Mtb* relies on phagocytes, primarily macrophages and dendritic cells, which engulf the bacteria and undergo metabolic reprogramming to produce pro-inflammatory cytokines and chemokines that recruit additional immune cells [2, 3]. These phagocytes also process and present *Mtb* antigens to T cells, which initiate adaptive immune responses, involving particularly CD4^+^ and CD8^+^ subsets, with the production of cytokines like IFN-γ to activate macrophages and enhance bacterial killing [4, 5]. One of the key anti-mycobacterial molecules produced by macrophages and other immune cells is nitric oxide (NO) [6, 7], which is synthesized from arginine catabolism by the inducible NO synthase 2 (Nos2) [8]. However, arginine is also catabolized by arginase enzymes, including cytosolic arginase-1 (Arg1) and mitochondrial arginase-2 (Arg2) [9, 10], which compete with Nos2 for the same substrate. Studies show that *Mtb* exploits this competition by upregulating Arg1 expression, thereby limiting NO production and impairing bacterial clearance [11]. Similarly, *Arg2* has been identified as one of the most prominent metabolic genes regulated by the interleukin (IL)-10/miR-155 axis and plays a critical role in shifting macrophages from a lipopolysaccharide (LPS)-induced inflammatory state toward an oxidative phenotype [10, 12]. Arg2 is also the predominant arginase isoform in dendritic cells (DCs), where it regulates NO production and T cell activation by controlling arginine availability [13]. Notably, during *Mtb* infection, *Arg2* is upregulated alongside *Nos2* in infected macrophages during early M1-like polarization and in the acute phase of lung infection in mice [14], although its precise role in TB pathogenesis remains unclear.

The dual roles of arginine metabolism in immune regulation make it an attractive target for host-directed therapies (HDTs) in TB [15]. Modulating arginine availability, either through supplementation or arginase inhibition, could theoretically enhance NO production and improve control of *Mtb*. However, clinical studies on arginine supplementation have yielded mixed results. While one study found no significant effects [16], another reported improved outcomes, including increased body mass index, reduced constitutional symptoms, and decreased C-reactive protein levels when arginine supplementation was combined with standard TB therapy, though the underlying immunological mechanisms remain speculative [17]. Meanwhile, pharmacological inhibition of arginase activity with CB1158, a small-molecule arginase inhibitor known to enhance anti-tumor immunity [18], has not yet been evaluated in the context of TB.

In this study, we investigated the effects of arginine supplementation and arginase inhibition during the acute phase of *Mtb* infection in a murine lung infection model [19]. Our findings reveal that arginine supplementation promotes an effective host defense by balancing proinflammatory responses with anti-inflammatory processes, whereas arginase inhibition disrupts this balance, resulting in compromised tissue repair and exacerbated lung pathology. These results underscore the critical role of arginase-mediated pathways in mitigating inflammation-induced tissue damage, promoting repair and homeostasis, and highlight the potential of arginine supplementation as an HDT strategy to improve TB treatment outcomes.

## Results and discussion

### Effects on the expression of arginine metabolism enzymes

**Fig. 1:**
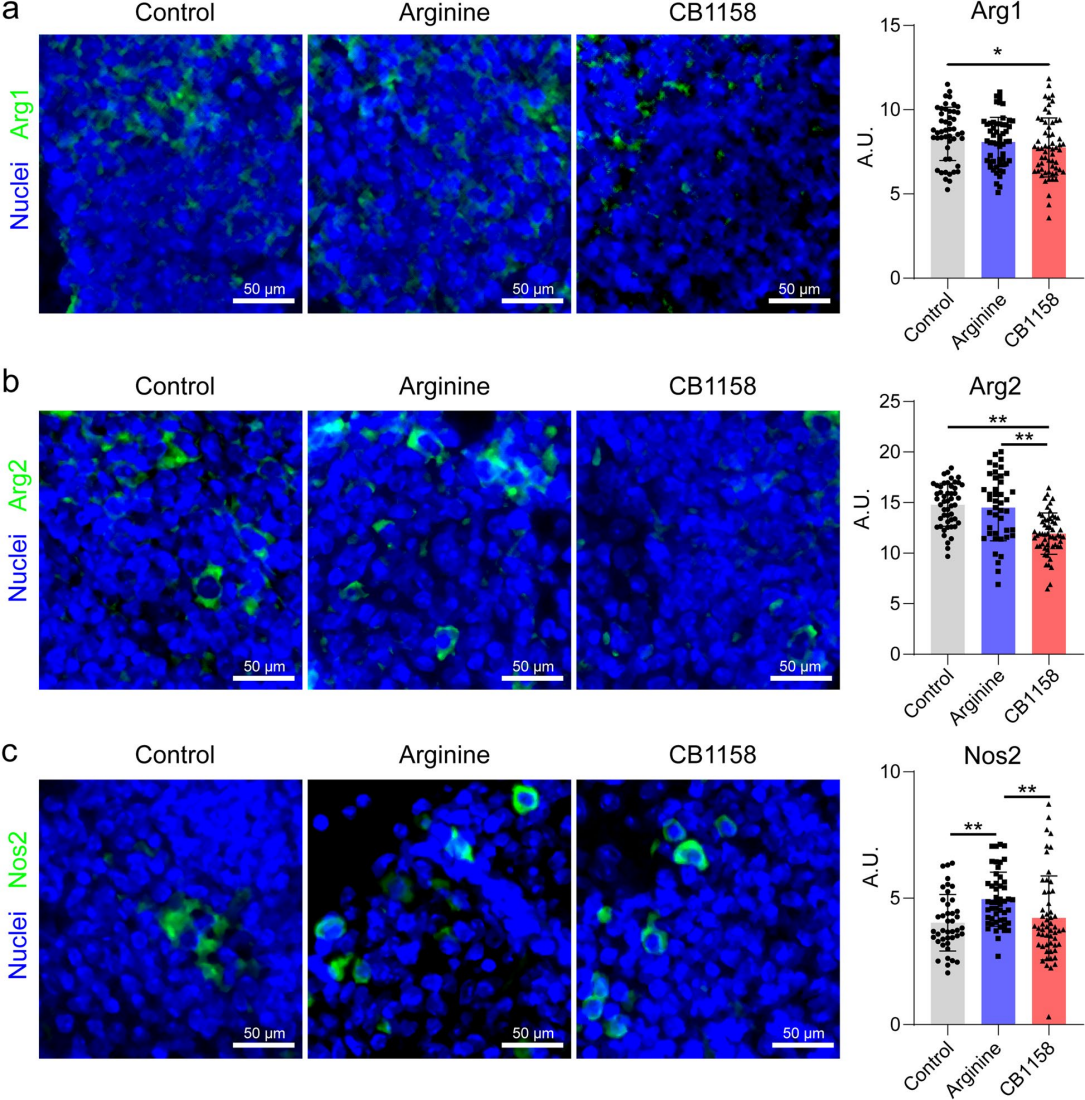
Effects of arginine supplementation and arginase inhibition on arginine metabolism enzymes in *Mycobacterium tuberculosis* (*Mtb*)-infected mouse lungs. C57BL/6 mice were aerosol-infected with ~ 50 CFU of *Mtb* H37Rv and treated daily via oral gavage with PBS (control), arginine (1.5 g/kg body weight), or the arginase inhibitor CB1158 (100 mg/kg body weight) from day 1 post-infection (p.i.) until day 35 p.i. At day 35 p.i., lung tissues were harvested, formalin-fixed, sectioned, and immunostained for Arg1 (**a**), Arg2 (**b**), and Nos2 (**c**). Representative images are shown (scale bar = 50 μm). Protein expression was quantified in 50–100 randomly selected granuloma-like regions per group (*n* = 4 mice per group). Data are presented as mean ± standard deviation. Significance between groups (arginine- or CB1158-treated vs. infection-only control, and arginine vs. CB1158-treated) was determined using a two-tailed Student’s *t*-test. *p < 0.05, **p < 0.01. A.U.: arbitrary unit

To assess the impact of arginine supplementation and arginase inhibition on key enzymes involved in arginine catabolism, we analyzed their expression in *Mtb*-infected mouse lungs. Immunofluorescence staining showed that arginine supplementation significantly increased the expression of Nos2 within granuloma-like regions but did not significantly alter the protein levels of Arg1 or Arg2 (Fig. 1a–c), compared to the infection-only control group. In contrast, arginase inhibition markedly reduced the expression of both Arg1 and Arg2 but had minimal effects on Nos2 protein levels (Fig. 1a–c).

Arginases and Nos2 are proposed to compete for the common substrate arginine, and decreased arginase would elevate Nos2 activity [20]. Our observation of no significant effects on Nos2 protein levels by CB1158 treatment aligns with studies that show arginase inhibition does not necessarily enhance Nos2 activity when there is a constant supply of arginine [21]. During infection, which is often accompanied by catabolic stress, arginine can be synthesized from precursors such as citrulline, glutamine, and proline, or derived from protein breakdown, ensuring its availability [22]. This continuous supply of arginine likely explains the lack of a significant impact of CB1158 on Nos2 protein levels in the lungs of infected mice. The observed upregulation of Nos2 following arginine supplementation supports previous findings that increased extracellular arginine enhances Nos2 function and NO production [23]. Given that NO plays a critical role in inhibiting *Mtb* growth [24, 25], and improving lung pathology by suppressing IL-1 and 12/15-lipoxygenase-driven neutrophil recruitment cascades [26, 27], these findings suggest the beneficial role of arginine supplementation in enhancing antimicrobial defense while mitigating tissue damage.

### Exacerbation of lung pathology by arginase inhibition

**Fig. 2:**
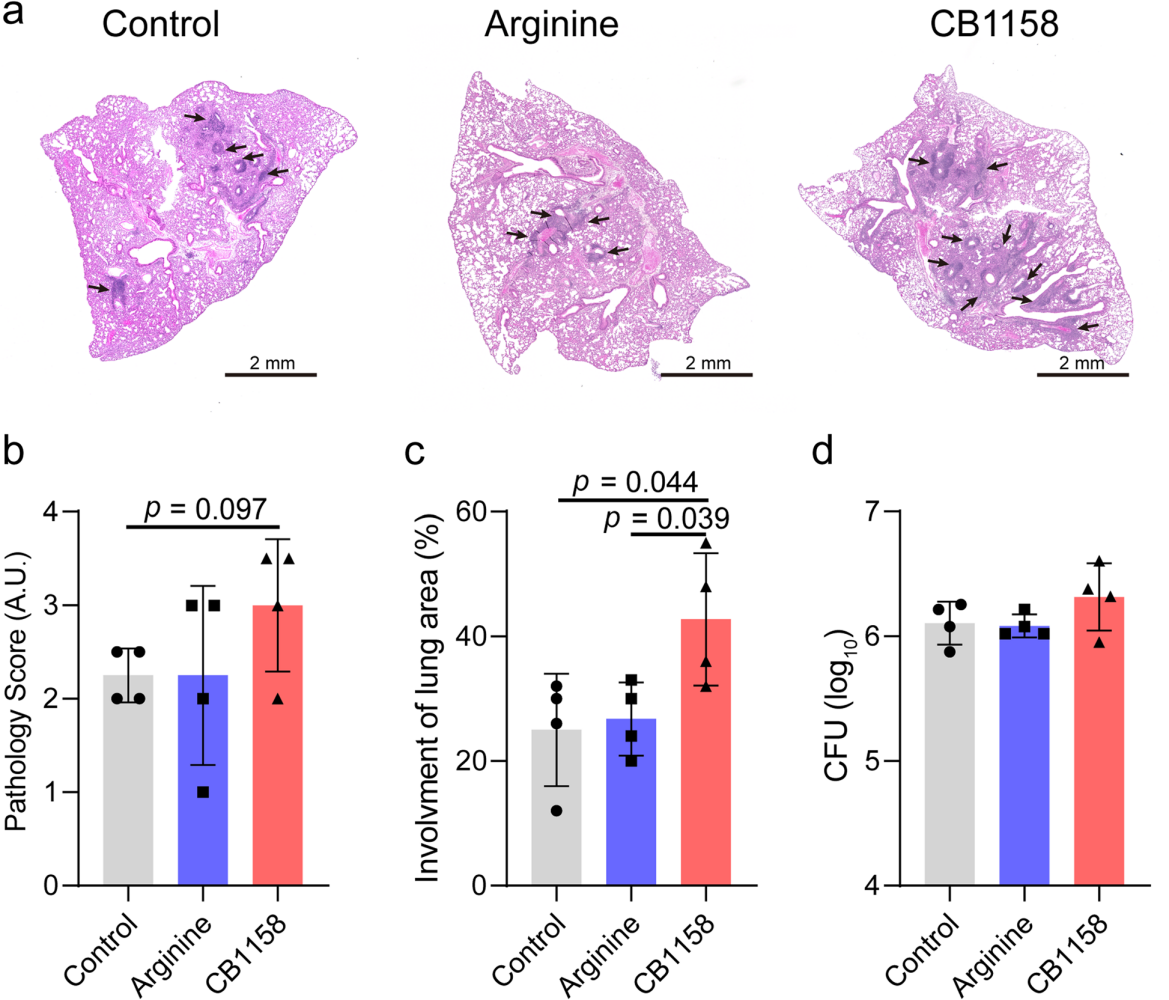
Arginase inhibition worsens lung pathology in infected mice. Lung sections from *Mtb*-infected C57BL/6 mice treated with PBS (control), arginine, or CB1158, as described in Fig. 1, were stained with hematoxylin and eosin (H&E). Representative H&E-stained images are shown, with arrows indicating regions of lung immune cell aggregates (lesions) (scale bar = 2 mm) (**a**). Lung pathology was evaluated semi-quantitatively (**b**, **c**). Bacillary load in the lungs of infected mice was measured at day 35 p.i. across groups (**d**). Data are represented as mean ± standard deviation (*n* = 4–5 mice per group). Statistical significance was determined between groups (arginine- or CB1158-treated vs. infection-only control, and arginine vs. CB1158-treated) using a two-tailed Student’s *t*-test. A *p* < 0.05 is considered statistically significant. A.U.: arbitrary unit

To assess the impact of treatments on disease pathology, we performed histopathological analysis of hematoxylin and eosin (H&E)-stained lung sections, using a modified scoring system that incorporates cellularity and granuloma architecture [28]. The two treatments had distinct effects on disease pathology (Fig. 2a–c). Relative to the infection-only group, arginine supplementation did not significantly alter lung pathology, as indicated by comparable pathological scores and granuloma area involvement (Fig. 2b, c). In contrast, treatment with the arginase inhibitor CB1158 exhibited a trend toward increased pathological scores (*p* = 0.097) (Fig. 2b) and significantly increased granuloma area involvement (*p* = 0.044) (Fig. 2c). Moreover, granuloma involvement was significantly greater in CB1158-treated mice than in those receiving arginine supplementation (*p* = 0.039) (Fig. 2c). These findings indicate that arginase inhibition worsens lung pathology, despite bacterial loads in the lungs remaining unaffected (Fig. 2d). The exacerbation of lung pathology following arginase inhibition is likely associated with disruption of critical pathways involved in resolving inflammation and/or promoting tissue repair. This highlights the potential risks of targeting arginases without fully understanding their context-dependent roles in host immunity and disease progression.

### Effects on lung immune cell population and function

**Fig. 3:**
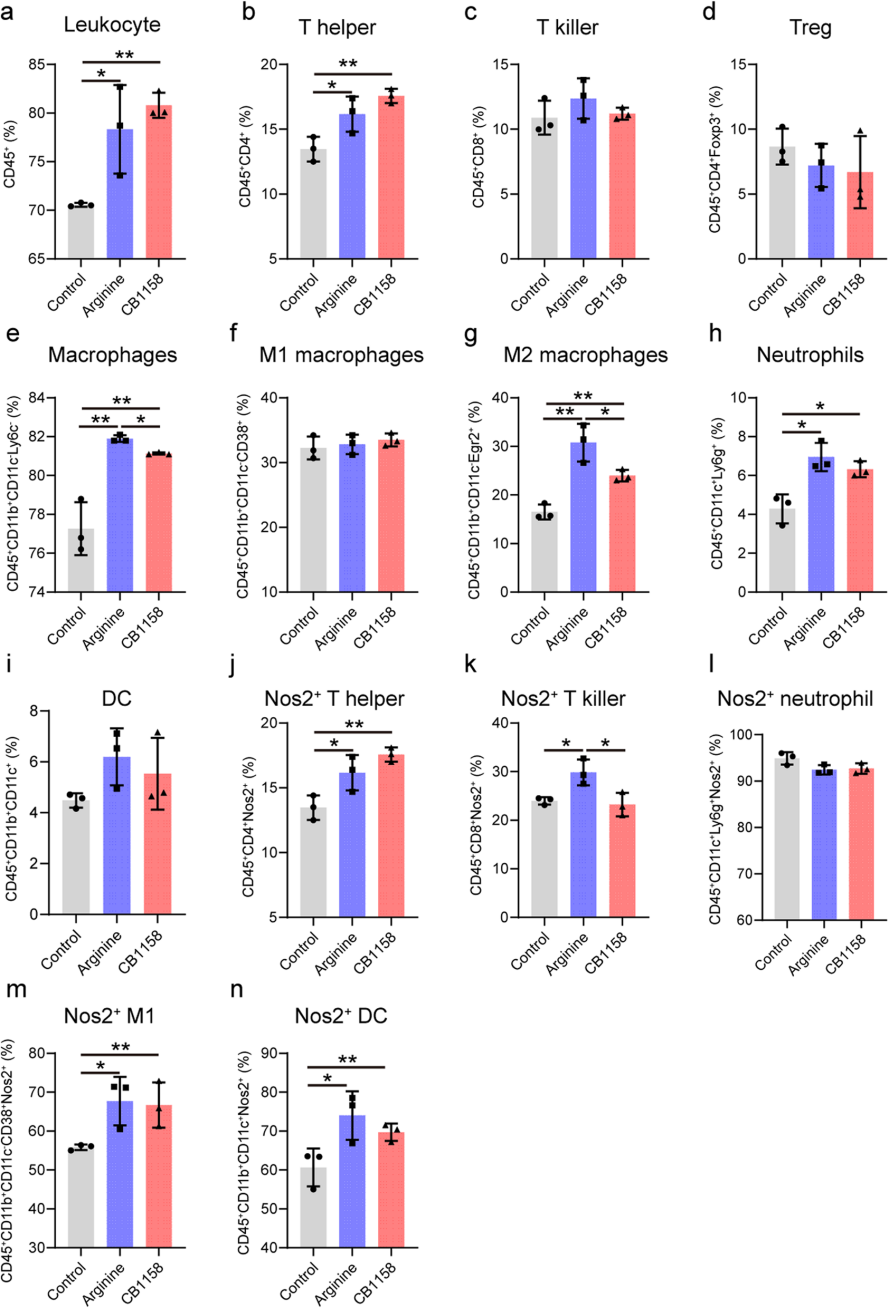
Changes of immune cell population and function by arginine supplementation and arginase inhibition in lungs of infected mice. C57BL/6 mice infected with *Mtb* and treated as described in Fig. 1 were sacrificed at day 35 p.i. Lung single-cell suspensions were prepared, and the proportions of immune cell subsets were analyzed by flow cytometry. Quantified populations included leukocytes (CD45^+^) (**a**), T helper cells (CD45^+^CD4^+^) (**b**), T killer cells (CD45^+^CD8^+^) (**c**), regulatory T cells (CD45^+^CD4^+^Foxp3^+^) (**d**), macrophages (CD45^+^CD11b^+^CD11c^−^Ly6c^−^) (**e**), M1 macrophages (CD45^+^CD11b^+^CD11c^−^CD38^+^) (**f**), M2 macrophages (CD45^+^CD11b^+^CD11c^−^Egr2^+^) (**g**), neutrophils (CD45^+^CD11c^+^Ly6g^+^) (**h**), and dendritic cells (CD45^+^CD11b^+^CD11c^+^) (**i**). Expression of Nos2 in T helper cells (**j**), T killer cells (**k**), neutrophils (**l**), M1 macrophages (**m**), and dendritic cells (**n**) was also assessed. Data were acquired on an LSRFortessa X-20 flow cytometer and analyzed using FlowJo software. Data represent mean ± standard deviation (*n* = 3 mice per group). Statistical significance was determined between groups (arginine- or CB1158-treated groups vs. infection-only control, and arginine vs. CB1158-treated) by a two-tailed Student’s *t*-test. *p < 0.05, **p < 0.01

We also performed flow cytometry analysis to characterize effects of arginine supplementation and arginase inhibitor on lung immune cell populations and their activation states. Both treatments resulted in a similar pattern of immune cells infiltration and activation relative to the infection-only control group. Leukocyte infiltration (Fig. 3a), including T helper cells (Fig. 3b), macrophages (Fig. 3e, g), and neutrophils (Fig. 3h), was significantly increased in both intervention groups. Additionally, both treatments promoted a predominantly pro-inflammatory and bactericidal immune profile, as indicated by enhanced Nos2 expression in T helper cells (Fig. 3j), M1 macrophages (Fig. 3m), and dendritic cells (Fig. 3n). This response was accompanied by an increased anti-inflammatory response, characterized by a rise in M2 macrophages (Fig. 3g). Notably, CB1158 treatment differentially affected lung immune cell composition and function by reducing total macrophage numbers (Fig. 3e), particularly the M2 subtype (Fig. 3h), compared to the arginine-supplemented group. This suggests that arginase inhibition disrupts the balance between pro-inflammatory and anti-inflammatory responses, potentially contributing to the exacerbated pathology observed in the lungs of CB1158-treated mice (Fig. 2c).

The observed shifts in immune cell profiles align with prior studies showing increased pro-inflammatory immune cell populations and activation markers during *Mtb* infection, which enhance antigen presentation and effector functions [29]. The rise in M2 macrophages underscores the importance of arginine metabolism in maintaining the balance between pro-inflammatory and anti-inflammatory responses. Increased M2 macrophages likely contributed to inflammation resolution and tissue repair by secreting anti-inflammatory cytokines such as IL-10 and transforming growth factor beta (TGF-β), and by maintaining mitochondrial function via oxidative phosphorylation and fatty acid oxidation that support their energy demands for synthesizing extracellular matrix components like collagen essential for tissue repair [30, 31].

Arg1 is well-established as an anti-inflammatory mediator [32], and its suppression by CB1158 likely amplified the proinflammatory response in arginase-inhibited lungs. The role of Arg2 appears to be more context dependent. Evidence suggests that Arg2 can promote pro-inflammatory responses through mechanisms such as mitochondrial reactive oxygen species (ROS) generation [33, 34], and mitochondrial dynamics (our unpublished observations). Additionally, Arg2-faciliated ornithine production in mitochondria [10], a precursor for polyamines essential for cell proliferation, further highlights its potential importance in promoting immune responses. Thus, CB1158-mediated inhibition of Arg2 may also have contributed to the elevated M2 macrophages in lungs of CB1158-treated mice, albeit to a lesser degree compared to the arginine-supplemented group. Collectively, our data indicate that arginine supplementation promotes a balanced pro- and anti-inflammatory host immunity that supports *Mtb* control, while arginase inhibition disrupts this immune homeostasis, leading to exacerbated tissue damage.

### Effects on the expression of cytokine genes

**Fig. 4:**
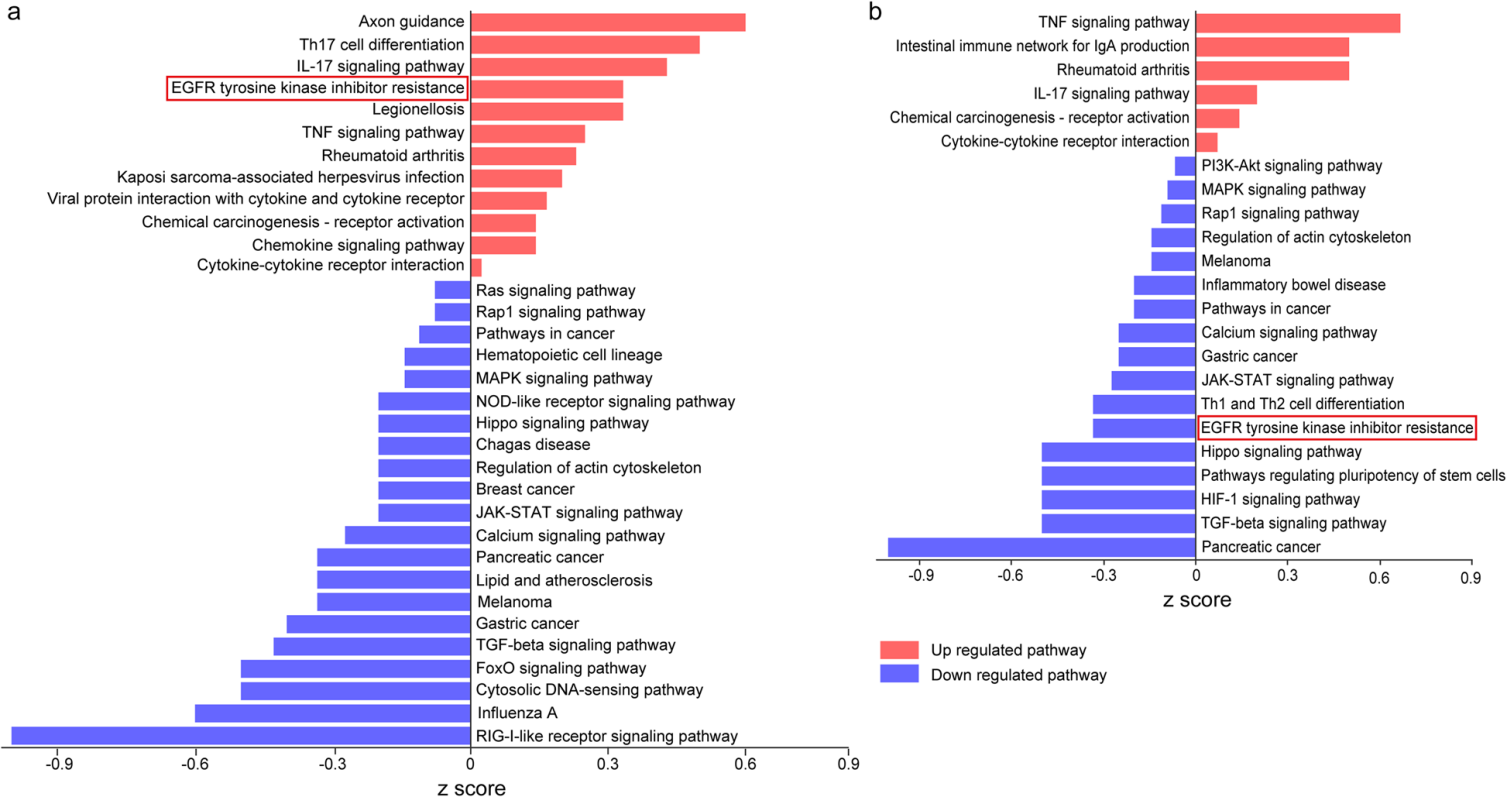
Effects of arginine supplementation and arginase inhibition on Kyoto Encyclopedia of Genes and Genomes (KEGG) cytokine signaling pathways in lungs of infected mice. C57BL/6 mice infected with *Mtb* and treated as described in Fig. 1 were sacrificed at day 35 p.i. Total RNA was extracted from lung tissues, reverse-transcribed to cDNA, and analyzed using a qPCR array containing 176 primer sets targeting cytokine genes (Mouse Cytokine Primer Library I and II). Gene expression levels were normalized to housekeeping genes, and differentially expressed cytokine genes in arginine- or CB1158-treated groups (compared to infection-only controls) were subjected to KEGG pathways enrichment analysis. Bar plots illustrate changes in KEGG pathways associated with arginine supplementation (**a**) and CB1158 treatment (**b**) compared to the infection-only control. Data represent analyses from 3 mice per group

To further elucidate the immunological effects of manipulating arginine metabolism by the two treatments, we analyzed cytokine gene expression profiles using the Mouse Cytokine Primer Library I and II (Real Time Primers, Elkins Park, PA, USA). Both arginine supplementation and CB1158 treatment altered cytokine expression profiles, resulting in enrichment of similar pathways based on the differentially expressed genes (DEGs) compared to the infection-only group (Fig. 4a, b). Notably, both interventions upregulated genes involved in the pro-inflammatory tumor necrosis factor (TNF) signaling pathway while concurrently downregulating genes associated with the anti-inflammatory TGF-β signaling pathway. However, a key difference emerged in their effects on the epidermal growth factor receptor (EGFR) tyrosine kinase inhibitor resistance pathway: arginine supplementation significantly upregulated this pathway, whereas CB1158 downregulated it (Fig. 4a, b, highlighted). This divergence was primarily driven by differential expression of the vascular endothelial growth factor A (*Vegfa*) gene, which showed a 9.18-fold increase with arginine supplementation but a 5.96-fold decrease following CB1158 treatment compared to the infection-only group (Table S1).

As a key regulator of angiogenesis and vascular permeability, Vegfa plays a crucial role in inflammatory processes, particularly in the recruitment of macrophages to granulomas through nonangiogenic mechanisms during mycobacterial infections [35]. Elevated serum vascular endothelial growth factor (VEGF) levels in patients with active pulmonary TB have been associated with reduced cavity formation and improved disease outcomes [36]. Furthermore, increased *Vegfa* expression, as observed with arginine supplementation, enhances angiogenesis, a process previously shown to be inducible by elevated extracellular arginine in a rat model [37]. Thus, the upregulation of *Vegfa* by arginine supplementation likely serves two key functions: promoting macrophage recruitment to granulomas to strengthen immune responses and mitigating tissue damage by maintaining angiogenesis. Conversely, the exacerbation of lung pathology with arginase inhibition may be attributed to the suppression of *Vegfa* expression, which could impair angiogenesis, destabilize granulomas, and hinder tissue repair mechanisms.

### Mitophagy disruption by arginase inhibition

**Fig. 5:**
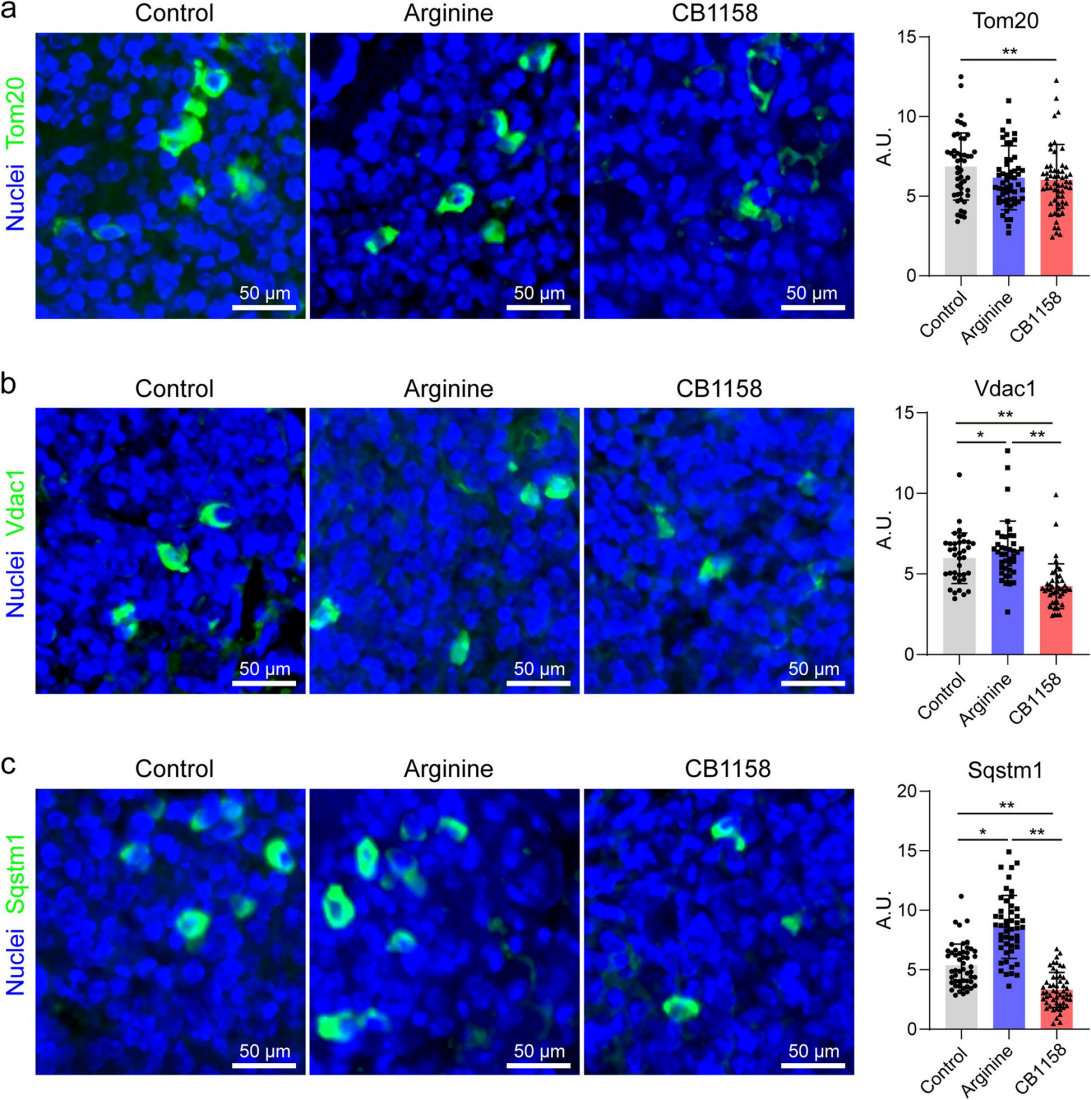
Differential effects of arginine supplementation and arginase inhibition on mitophagy protein expression in lungs of infected mice. Lung sections from *Mtb*-infected C57BL/6 mice treated with PBS (control), arginine, or CB1158, as described in Fig. 1, were immunostained for key proteins involved in the mitophagy pathway: Tom20 (**a**); Vdac1 (**b**); and Sqstm1 (**c**). Representative images of stained sections are shown (scale bars = 50 μm). Quantitative analysis of protein expression was performed in 50–100 randomly selected granuloma-like regions per group (*n* = 4–5 mice per group). Data are presented as mean ± standard deviation. Statistical significance between groups (arginine- or CB1158-treated vs. infection-only control, and arginine vs. CB1158-treated) was evaluated using a two-tailed Student’s *t*-test. *p < 0.05, **p < 0.01. A.U.: arbitrary unit

Given that arginine metabolism regulates NO production, polyamine synthesis, and energy metabolism, processes that influence mitochondrial function and autophagy-related pathways, we further investigated whether the differential effects of arginine supplementation and arginase inhibition were associated with mitophagy-mediated pathways and mitochondrial function. Immunofluorescence analysis was performed to assess mitophagy-related proteins, including ubiquitinated Vdac1 (a mitochondrial channel protein) and Tom20 (a key component of the translocase of the outer mitochondrial membrane), which serve as signals for mitophagy receptors such as Sequestosome 1 (Sqstm1 or p62) to recognize and clear damaged mitochondria [38–40]. Our analysis revealed that arginase inhibition by CB1158 significantly decreased the expression of Tom20, Vdac1 and Sqstm1 in granuloma-like regions of infected mouse lungs compared to the infection-only control group (Fig. 5a–c). In contrast, arginine supplementation significantly upregulated Sqstm1 and Vdac1 expression (Fig. 5b, c). These findings suggest that arginine supplementation enhances mitophagy through promoting key steps in the pathway, whereas arginase inhibition disrupts mitochondrial quality control by impairing these processes.

Mitophagy is essential for maintaining cellular homeostasis and immune defense during infection, as it facilitates the clearance of damaged mitochondria and reduces ROS production [41–43]. Polyamines, which are derived from ornithine, a product of arginase-mediated metabolism, are known to stabilize mitochondrial membranes and regulate autophagy [44]. The reduced expression of Sqstm1 observed following CB1158 treatment may be linked to diminished activity of mitochondrial Arg2, a key contributor to polyamine synthesis [45]. Polyamines such as putrescine, spermidine, and spermine play crucial roles in maintaining mitochondrial integrity and membrane potential [46]. Impaired polyamine availability due to arginase inhibition could account for decreased levels of Tom20 and Vdac1, further compromising mitophagy and mitochondrial function. Conversely, the upregulation of Vdac1 and Sqstm1 with arginine supplementation may reflect the role of arginine as a key regulator of the mTOR pathway [47], which orchestrates autophagy and mitochondrial dynamics. By sustaining mTOR activation, arginine supplementation supports mitochondrial renewal and enhances mitophagy. This process reduces mitochondrial ROS production, which can mitigate oxidative stress and tissue damage, while also facilitating the clearance of intracellular pathogens [48].

Overall, our findings indicate that arginine supplementation enhances mitophagy as part of its protective immune response during *Mtb* infection, likely through arginase-mediated pathways that support mitochondrial function and cellular homeostasis. In contrast, the disruption of these pathways by arginase inhibition highlights the critical role of balanced arginine metabolism in maintaining mitochondrial quality control and immune defense.

## Conclusion

This study highlights the critical role of arginine metabolism in regulating immune responses, lung pathology, and mitochondrial function during *Mtb* infection. The Nos2- and arginase-mediated pathways exhibit distinct but complementary functions, coordinating proinflammatory and anti-inflammatory responses to maintain immune cell integrity and function. Arginine supplementation demonstrated its potential to enhance protective immune responses by promoting Nos2 expression and driving proinflammatory activity, while simultaneously supporting angiogenesis and tissue repair through anti-inflammatory processes such as mitophagy, M2 macrophage activation, and *Vegfa* upregulation. Conversely, findings from arginase inhibition underscore the indispensable role of arginase-mediated pathways in maintaining immune homeostasis, with disruptions leading to exacerbated lung pathology and impaired mitochondrial quality control. In summary, these findings suggest that arginine supplementation holds promise as an adjunctive therapy for TB management. By balancing inflammation and tissue repair, arginine supplementation may help optimize host immunity while mitigating lung damage. Future studies should focus on delineating the long-term effects of arginine supplementation, particularly during the chronic stages of *Mtb* infection, and its potential synergistic benefits when combined with conventional antibiotic therapies.

## Materials and methods

### Animals

Eight-week-old female C57BL/6 mice were purchased from the Jackson Laboratory (Bar Harbor, ME, USA) and housed in a controlled environment at a constant temperature (20 ± 2 °C) under a 12-h light–dark cycle with ad libitum access to food and water. All animal procedures were performed in accordance with the Guide for the Care and Use of Laboratory Animals (National Institutes of Health). Experimental protocols were approved by the Institutional Animal Care and Use Committee (IACUC) at Rutgers University (protocol no. PROTO999900960). The Public Health Research Institute (PHRI) animal facility at Rutgers University is accredited by the Association for Assessment and Accreditation of Laboratory Animal Care (AAALAC) and adheres to the Animal Welfare Act (AWA) and Public Health Service Research Extension Act (PHSREA) and all other policies administered by the United States Department of Agriculture (USDA).

### Bacterial culture, aerosol infection, and mouse treatments

*Mtb* H37Rv was obtained from American Type Culture Collection (ATCC) [49], and cultured in Dubos Tween Albumin (DTA) medium [50] (Becton, Dickinson, Franklin Lakes, NJ, USA) at 37 °C to mid-log phase (OD_580_ nm = 0.3 to 0.5) and frozen in 1 ml of aliquots at −80 °C. Mice (*n* = 4–5/group) were aerosol infected with ~ 50 CFU of *Mtb* using a Glas-Col airborne infection system, as described previously [51]. Infection was verified by sacrificing three mice on day 1 post-infection (p.i.) to determine lung CFU inoculum. Treatments, including phosphate-buffered saline (PBS; control), arginine (1.5 g/kg body weight, once daily), or the arginase inhibitor CB1158 (100 mg/kg body weight, once daily), were administered via oral gavage starting day 1 p.i. and continued until day 35 p.i. At day 35 p.i., lung tissues from 4–5 mice per group were harvested. Portions of the lungs were homogenized in 1 X PBS with 0.05% Tween 20 (PBST, pH = 7.4) (Fisher Scientific, Lenexa, KS, USA) and 10-fold dilution series of lung lysate were plated on Middlebrook 7H10 agar plates (Becton, Dickinson, Franklin Lakes, NJ, USA) for CFU determination. The other portions of lungs were fixed in 10% neutral buffered formalin (Sigma, Saint Louis, MO, USA) for histological and immunofluorescence analysis, snap-frozen in liquid nitrogen and kept at kept at 80 °C for RNA isolation and qPCR array of cytokine gene expression, or used to generate single cell suspension for flow cytometry analysis.

### Flow cytometry analysis of lung immune cell populations and activation

Single cell suspensions from infected lungs were prepared using the Lung Dissociation Kit (Miltenyi Biotec, Bergisch Gladbach, Germany) and the gentleMACS™ Dissociator (Miltenyi Biotec, Bergisch Gladbach, Germany). Approximately one million cells were resuspended in FACS buffer (PBS + 1% FBS). Cells were stained first with the LIVE/DEAD Fixable Dead Cell Stains (Thermo Fisher Scientific, Waltham, MA, USA), followed by other cell surface markers with directly conjugated antibodies: anti-mouse CD45 (clone 30-F11, BD Pharmingen, San Diego, CA, USA), anti-mouse CD4 (clone GK1.5, BD Pharmingen, San Diego, CA, USA), anti-mouse CD8a (clone 53–6.7, BD Pharmingen, San Diego, CA, USA), anti-mouse CD38 (clone Ab90, BD Pharmingen, San Diego, CA, USA), anti-mouse CD11b (clone M1/70, BD Pharmingen, San Diego, CA, USA), anti-mouse CD11c (clone HL3, BD Pharmingen, San Diego, CA, USA), anti-mouse Ly6C (clone AL-21, BD Pharmingen, San Diego, CA, USA), anti-mouse Ly6G (clone 1A8, BD Pharmingen, San Diego, CA, USA). Following the staining of surface markers, cells were washed in FACS buffer, fixed and permeabilized in the Foxp3/Transcription Factor Staining Buffer Set (eBioscience, San Diego, CA, USA). Intracellular markers, including Foxp3, Nos2, and Egr2, were stained by antibodies of Anti-mouse Foxp3 (clone MF23, BD Pharmingen, San Diego, CA, USA), anti-mouse Nos2 (clone CXNFT, eBioscience, San Diego, CA, USA), and anti-mouse Egr2 (clone erongr2, eBioscience, San Diego, CA, USA). Data were acquired using an LSRFortessa X-20 (BD Biosciences, San Diego, CA, USA) and analyzed in FlowJo software (v10.6.2, Tree Star, Inc., Ashland, OR, USA) using fluorescence-minus-one controls for gating.

### Analysis of lung pathology and protein immunofluorescence

Formalin-fixed, paraffin-embedded lung sections (7 µm thick) were stained with H&E for histological evaluation [52] and imaged with an automated digital widefield microscope on a BioTek Cytation 5 (Agilent, Santa Clara, CA, USA). Morphometric analysis of the percentage of lung area involved in disease pathology was performed using Sigmascan Pro Software (version 5.0, Systat Softwares, Inc., San Jose, CA, USA). For protein expression analysis by immunofluorescence, lung sections were stained with antibodies targeting Nos2 (1:500, 13120S**,** Cell Signaling, Danvers, MA, USA), Arg1 (1:500, ab212522, Abcam, Waltham, MA, USA), Arg2 (1:500**,** ab228700, Abcam, Waltham, MA, USA), Sqstm1/p62 (1:500, Cat.No: 88588S, Cell Signaling, Danvers, MA, USA), Tom20/Tomm20 (1:500, MABT166, Sigma-Aldrich, Burlington, MA, USA), and Vdac1, (1:1000, SAB5201374-100UG, Sigma-Aldrich, Burlington, MA, USA). Nuclei were counterstained with 4′,6-diamidino-2-phenylindole (DAPI) (1 µg/mL). Images were acquired using an Axiovert 200 M widefield epifluorescence microscope (Zeiss, Oberkochen, Germany) controlled by MetaMorph software (version 7.0, Molecular Devices, San Jose, CA, USA), as previously described [53]. Antibody specificity and autofluorescence were confirmed by replacing the primary antibody with a non-specific myeloma protein of the same isotype. The expression levels of target proteins were analyzed within randomly selected regions of interest (ROIs) located in the granuloma-like area of the infected lungs. These areas were identified in the DAPI channel by their characteristic morphology, with compressed and aggregated nuclei surrounded by healthy alveolar tissue. Quantitative expression analysis of each target protein was performed by measuring the intensity of positive pixels within the selected ROIs (Fig. S1). For each group, 50–100 ROIs from sections of 4–5 mice were analyzed. ROIs were selected based on DAPI staining to ensure unbiased sampling.

### qPCR array of cytokine gene expression and KEGG pathway analysis

Frozen lungs were disrupted with a Mini Bead Mill Homogenizer (VWR, Radnor, PA, USA). Total RNA was extracted with RNAzol® RT Column Kit (Molecular Research Center, Cincinnati, OH, USA), following the manufacturer’s instructions. cDNA was synthesized using the QuantiTect Reverse Transcription Kit (Invitrogen, Waltham, MA, USA). qPCR was performed using Power SYBR™ Green PCR Master Mix (Waltham, MA, USA) on a AriaMx System (Agilent, Santa Clara, CA, USA) under the following conditions: primer concentration of 0.1 µM, with a thermal cycling program of 95 °C for 10 s followed by 58 °C for 45 s for 50 cycles, as per the manufacturer’s instructions. A total of 176 primer sets directed against cytokines (realtimeprimers, Melrose Park, PA, USA) were used to characterize the immune response of lungs in each group (Tables S2 & S3). Data were normalized to housekeeping genes, according to the manufacturer’s instructions (available at https://pcrarray.com) [54]. Briefly, relative gene expression was calculated by determining ΔCt values, using the difference between the cycle threshold (Ct) value of the gene of interest and the mean Ct value of the three most stable housekeeping genes: *Actb*, *Ppi*, and *Gapdh*. The differential analysis was carried out by comparing the relative expression of genes between arginine treated or CB1158 treated groups versus infection-only control group. The DEGs (Table S1) were subjected to Kyoto Encyclopedia of Genes and Genomes (KEGG, http://www.genome.jp/kegg/) enrichment analyses [55], using the ClusterProfiler package in R (version 4.3.2).

### Statistical analysis

Comparisons between groups were performed using unpaired, two-tailed Student’s t-tests with the GraphPad Prism 8.0 (GraphPad Software, Boston, MA, USA). Data are presented as mean ± standard deviation in all figures. Statistical significance was indicated by *p* < 0.05 and represented as follows: * *p* < 0.05, ** *p* < 0.01.

## Supplementary Information

Supplementary Material 1.

## Data Availability

Additional data are provided in the Supplementary files, including the definition of the region of interest (ROI) for lung immune cell aggregates (granuloma-like regions) (Fig. S1), genes of the mouse cytokine and chemokine library I and II (Tables S2 & S3), and fold-change values of gene expression by quantitative RT-PCR (Table S1).
