# Supplementary material for: Arginine as host directed therapy in tuberculosis: insights from modulating arginine metabolism by supplementation and arginase inhibition
Source: One Health Adv. 2025 Mar 21;3(1):5. doi: 10.1186/s44280-025-00070-6 (PMC11928424; doi:10.1186/s44280-025-00070-6)
Supplement: Supplementary file 1 — Supplementary Material 1. [file 44280_2025_70_MOESM1_ESM.docx]

**SUPPLEMENTARY INFORMATION FOR**

**Arginine as host directed therapy in tuberculosis: insights from modulating arginine metabolism by supplementation and arginase inhibition**

Qingkui Jiang^1^, Ranjeet Kumar^1^, Yi Zhao^2,3^, Selvakumar Subbian^1^, Lanbo Shi^1^*

^1^ Public Health Research Institute, New Jersey Medical School, Rutgers Biomedical and Health Sciences, Rutgers, The State University of New Jersey, Newark, 07103, New Jersey, USA

^2^ Guangdong Provincial Key Laboratory of Medical Molecular Diagnostics, The First Dongguan Affiliated Hospital, Guangdong Medical University, Dongguan, Guangdong 523713, China

^3^ Microbiology and Immunology Department, Guangdong Medical University, Dongguan, Guangdong 523808, China

*Correspondence: Lanbo Shi

email: [shila@njms.rutgers.edu](mailto:shila@njms.rutgers.edu)

This supplementary document includes

Figure **S1** and Tables **S1–S3**


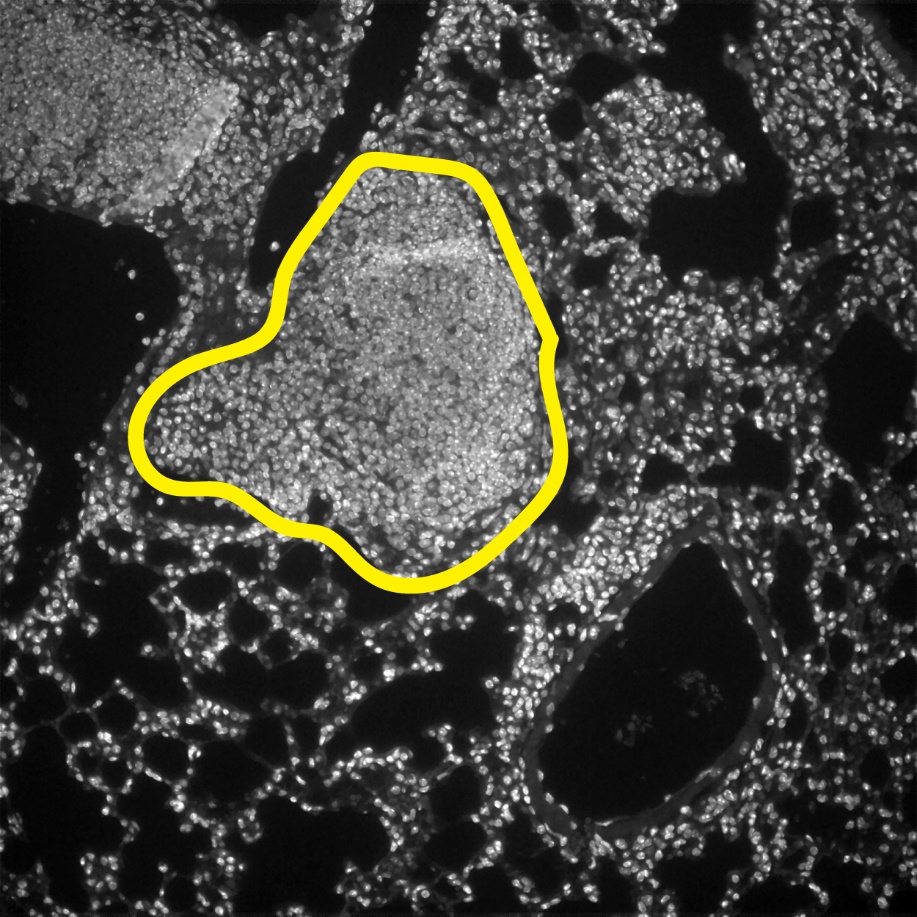


**Figure S1. Representative region of interest (ROI) for protein expression analysis in lung immunofluorescence images from *Mtb*-infected mice**

The selected ROI highlights a granuloma-like region in the lungs of *Mtb*-infected mice, characterized by dense cellular aggregation and disrupted lung architecture. Tissue sections were stained with DAPI to visualize nuclear structures.

**Table S1. Differential expression of cytokine genes in response to arginine supplementation and CB1158 treatment compared to the infection-only control**

| **Arginine vs. control** | | **CB1158 vs. control** | |
| --- | --- | --- | --- |
| Gene  Symbol | Fold  Change | Gene  Symbol | Fold  Change |
| *Efnb3* | 13.00 | *Gdf7* | 13.63 |
| *Csf3* | 12.93 | *Efnb3* | 12.55 |
| *Vegfa* | 9.18 | *Mstn* | 9.55 |
| *Clc* | 8.86 | *Cxcl2* | 9.07 |
| *Mstn* | 8.73 | *Il9* | 6.75 |
| *Il9* | 8.43 | *Il15* | 6.42 |
| *Gdf7* | 6.91 | *Csf1* | 5.98 |
| *Fgf23* | 6.59 | *Tnfsf11* | 5.59 |
| *Cxcl2* | 6.24 | *Nrg3* | 5.15 |
| *Il17f* | 6.23 | *Clc* | 4.56 |
| *Cxcl12* | 6.07 | *Fgf5* | 4.32 |
| *Csf1* | 5.69 | *Il1f5* | 4.30 |
| *Efna3* | 4.84 | *Il2* | 3.36 |
| *Il2* | 4.73 | *Il6* | 3.02 |
| *Il15* | 4.29 | *Cxcl13* | 2.74 |
| *Bmp15* | 3.96 | *Fgf4* | 2.70 |
| *Bmp10* | 3.91 | *Bdnf* | 2.66 |
| *Il1f8* | 3.90 | *Bmp3* | 2.20 |
| *Cd70* | 3.86 | *Cd70* | 2.16 |
| *Tnfsf11* | 3.64 | *Inhba* | 2.15 |
| *Fgf13* | 3.32 | *Cxcl5* | 2.13 |
| *Il6* | 3.11 | *Fgf9* | 2.05 |
| *Tnfsf4* | 3.03 | *Tgfb2* | –2.34 |
| *Cxcl5* | 2.88 | *Il1f6* | –2.40 |
| *Bmp8a* | 2.55 | *Fgf20* | –2.40 |
| *Fgf4* | 2.22 | *Bmp15* | –2.53 |
| *Gh* | 2.02 | *Ifnb1* | –2.54 |
| *Fgf7* | 2.01 | *Bmp8b* | –2.80 |
| *Ccl28* | 2.01 | *Lefty1* | –3.02 |
| *Tnfsf12* | –2.03 | *Ifne1* | –3.25 |
| *Fgf20* | –2.16 | *Egf* | –3.64 |
| *Ccl8* | –2.19 | *Il4* | –3.66 |
| *Tnfsf10* | –2.20 | *Fgf14* | –3.70 |
| *Gdf10* | –2.24 | *Inhbb* | –3.93 |
| *Ccl5* | –2.27 | *Amh* | –4.54 |
| *Il18* | –2.47 | *Fgf23* | –4.80 |
| *Bmp6* | –2.47 | *Tpo* | –5.13 |
| *Nodal* | –2.51 | *Fgf21* | –5.17 |
| *Fgf15* | –2.84 | *Vegfa* | –5.96 |
| *Il1f6* | –3.05 | *Ccl24* | –6.01 |
| *Tgfb2* | –3.18 | *Fgf13* | –6.28 |
| *Ifnk* | –3.26 | *Epo* | –7.03 |
| *Il28* | –3.35 | *Lefty2* | –8.36 |
| *Nrg4* | –3.45 | *Lep* | –9.93 |
| *Il11* | –3.79 | *Il13* | –11.22 |
| *Ifnb1* | –4.19 |  |  |
| *Ccl24* | –4.48 |  |  |
| *Fgf16* | –5.82 |  |  |
| *Csf2* | –5.85 |  |  |
| *Il4* | –6.39 |  |  |
| *Epo* | –6.49 |  |  |
| *Fgf1* | –6.63 |  |  |
| *Fgf21* | –6.80 |  |  |
| *Lep* | –7.57 |  |  |
| *Lefty2* | –8.02 |  |  |
| *Vegfb* | –8.11 |  |  |
| *Ifne1* | –9.34 |  |  |
| *Efnb2* | -9.43 |  |  |
| *Inhbb* | –14.79 |  |  |
| *Egf* | –16.52 |  |  |

**Table S2. Mouse Cytokine Primer Library I**

| Well | Symbol | Name | Well | Symbol | Name |
| --- | --- | --- | --- | --- | --- |
| A1 | *Il2* | interleukin 2 | E1 | *Fgf1* | fibroblast growth factor 1 |
| A2 | *Il3* | interleukin 3 | E2 | *Fgf2* | fibroblast growth factor 2 |
| A3 | *Il4* | interleukin 4 | E3 | *Fgf3* | fibroblast growth factor 3 |
| A4 | *Il7* | interleukin 7 | E4 | *Fgf4* | fibroblast growth factor 4 |
| A5 | *Il9* | interleukin 9 | E5 | *Fgf5* | fibroblast growth factor 5 |
| A6 | *Il11* | interleukin 11 | E6 | *Fgf6* | fibroblast growth factor 6 |
| A7 | *Il13* | interleukin 13 | E7 | *Fgf7* | fibroblast growth factor 7 |
| A8 | *Il15* | interleukin 15 | E8 | *Fgf8* | fibroblast growth factor 8 |
| A9 | *Il21* | interleukin 21 | E9 | *Fgf9* | fibroblast growth factor 9 |
| A10 | *Tslp* | thymic stromal lymphopoietin | E10 | *Fgf10* | fibroblast growth factor 10 |
| A11 | *Il5* | interleukin 5 | E11 | *Fgf11* | fibroblast growth factor 11 |
| A12 | *Csf2* | colony stimulating factor 2 | E12 | *Fgf12* | fibroblast growth factor 12 |
| B1 | *Il6* | interleukin 6 | F1 | *Fgf13* | fibroblast growth factor 13 |
| B2 | *Il12a* | interleukin 12a | F2 | *Fgf14* | fibroblast growth factor 14 |
| B3 | *Il12b* | interleukin 12b | F3 | *Fgf15* | fibroblast growth factor 15 |
| B4 | *Il23a* | interleukin 23, alpha subunit p19 | F4 | *Fgf16* | fibroblast growth factor 16 |
| B5 | *Osm* | oncostatin M | F5 | *Fgf17* | fibroblast growth factor 17 |
| B6 | *Lif* | leukemia inhibitory factor | F6 | *Fgf18* | fibroblast growth factor 18 |
| B7 | *Zfp91* | zinc finger protein 91 | F7 | *Fgf20* | fibroblast growth factor 20 |
| B8 | *Clc* | cardiotrophin-like cytokine factor 1 | F8 | *Fgf21* | fibroblast growth factor 21 |
| B9 | *Ctf1* | cardiotrophin 1 | F9 | *Fgf22* | fibroblast growth factor 22 |
| B10 | *Csf3* | colony stimulating factor 3 | F10 | *Fgf23* | fibroblast growth factor 23 |
| B11 | *Lep* | leptin | F11 | *Nrg1* | neuregulin 1 |
| B12 | *Epo* | erythropoietin | F12 | *Nrg3* | neuregulin 3 |
| C1 | *Gh* | growth hormone | G1 | *Nrg4* | neuregulin 4 |
| C2 | *Prl* | prolactin | G2 | *Igf1* | insulin-like growth factor 1 |
| C3 | *Tpo* | thyroid peroxidase | G3 | *Hgf* | hepatocyte growth factor |
| C4 | *Ifnb1* | interferon beta 1 | G4 | *Egf* | epidermal growth factor |
| C5 | *Ifnk* | interferon kappa precursor | G5 | *Efna1* | ephrin A1 |
| C6 | *Ifne1* | interferon epsilon 1 | G6 | *Efna2* | ephrin A2 |
| C7 | *Il28* | interleukin 28 | G7 | *Efna3* | ephrin A3 |
| C8 | *Ifng* | interferon gamma | G8 | *Efna4* | ephrin A4 |
| C9 | *Il10* | interleukin 10 | G9 | *Efna5* | ephrin A5 |
| C10 | *Il19* | interleukin 19 | G10 | *Efnb1* | ephrin B1 |
| C11 | *Il20* | interleukin 20 | G11 | *Efnb2* | ephrin B2 |
| C12 | *Il24* | interleukin 24 | G12 | *Efnb3* | ephrin B3 |
| D1 | *Vegfa* | vascular endothelial growth factor A | H1 | *Gas6* | growth arrest specific 6 |
| D2 | *Vegfb* | vascular endothelial growth factor B | H2 | *Angpt1* | angiopoietin 1 |
| D3 | *Vegfc* | vascular endothelial growth factor C | H3 | *Angpt2* | angiopoietin 2 |
| D4 | *Figf* | c-fos induced growth factor | H4 | *Angpt4* | angiopoietin 4 |
| D5 | *Pdgfc* | platelet-derived growth factor, C polypeptide | H5 | *Actb* | Actin, beta |
| D6 | *Pdgfd* | platelet-derived growth factor, D polypeptide | H6 | *B2m* | Beta-2 microglobulin |
| D7 | *Ngfb* | nerve growth factor, beta | H7 | *Gapd* | glyceraldehyde-3-phosphate dehydrogenase |
| D8 | *Ntf3* | neurotrophin 3 | H8 | *Gusb* | Glucuronidase, beta |
| D9 | *Ntf5* | neurotrophin 5 | H9 | *Hprt1* | Hypoxanthine guanine phosphoribosyl transferase 1 |
| D10 | *Bdnf* | brain derived neurotrophic factor | H10 | *Pgk1* | Phosphoglycerate kinase 1 |
| D11 | *Gdnf* | glial cell line derived neurotrophic factor | H11 | *Ppia* | Peptidylprolyl isomerase A |
| D12 | *Csf1* | colony stimulating factor 1 | H12 | *Rpl13a* | Ribosomal protein L13a |

**Table S3: Mouse Cytokine Primer Library II**

| Well | Symbol | Name | Well | Symbol | Name |
| --- | --- | --- | --- | --- | --- |
| A1 | *Tnf* | tumor necrosis factor | E1 | *Inhbc* | inhibin beta-C |
| A2 | *Lta* | lymphotoxin A | E2 | *Inhbe* | inhibin beta-E |
| A3 | *Ltb* | lymphotoxin B | E3 | *Tgfb1* | transforming growth factor, beta 1 |
| A4 | *Tnfsf4* | tumor necrosis factor (ligand) superfamily, member 4 | E4 | *Tgfb2* | transforming growth factor, beta 2 |
| A5 | *Cd40lg* | CD40 ligand | E5 | *Tgfb3* | transforming growth factor, beta 3 |
| A6 | *Fasl* | Fas ligand (TNF superfamily, member 6) | E6 | *Amh* | anti-Mullerian hormone |
| A7 | *Cd70* | CD70 antigen | E7 | *Inha* | inhibin alpha |
| A8 | *Tnfsf8* | tumor necrosis factor (ligand) superfamily, member 8 | E8 | *Bmp1* | bone morphogenetic protein 1 |
| A9 | *Tnfsf9* | tumor necrosis factor (ligand) superfamily, member 9 | E9 | *Bmp10* | bone morphogenetic protein 10 |
| A10 | *Tnfsf10* | tumor necrosis factor (ligand) superfamily, member 10 | E10 | *Gdf15* | growth differentiation factor 15 |
| A11 | *Tnfsf11* | tumor necrosis factor (ligand) superfamily, member 11 | E11 | *Lefty1* | left right determination factor 1 |
| A12 | *Tnfsf12* | tumor necrosis factor (ligand) superfamily, member 13 | E12 | *Lefty2* | Left-right determination factor 2 |
| B1 | *Tnfsf13b* | tumor necrosis factor (ligand) superfamily, member 13b | F1 | *Ccl2* | chemokine (C-C motif) ligand 2 |
| B2 | *Tnfsf14* | tumor necrosis factor (ligand) superfamily, member 14 | F2 | *Ccl3* | chemokine (C-C motif) ligand 3 |
| B3 | *Tnfsf15* | tumor necrosis factor (ligand) superfamily, member 15 | F3 | *Ccl4* | chemokine (C-C motif) ligand 4 |
| B4 | *Tnfsf18* | tumor necrosis factor (ligand) superfamily, member 18 | F4 | *Ccl5* | chemokine (C-C motif) ligand 5 |
| B5 | *Eda* | ectodysplasin-A | F5 | *Ccl7* | chemokine (C-C motif) ligand 7 |
| B6 | *Il1a* | interleukin 1 alpha | F6 | *Ccl8* | chemokine (C-C motif) ligand 8 |
| B7 | *Il1b* | interleukin 1 beta | F7 | *Ccl11* | chemokine (C-C motif) ligand 11 |
| B8 | *Il1rn* | interleukin 1 receptor antagonist | F8 | *Ccl17* | chemokine (C-C motif) ligand 17 |
| B9 | *Il18* | interleukin 18 | F9 | *Ccl19* | chemokine (C-C motif) ligand 19 |
| B10 | *Il1f5* | interleukin 1 family, member 5 (delta) | F10 | *Ccl20* | chemokine (C-C motif) ligand 20 |
| B11 | *Il1f6* | interleukin 1 family, member 6 | F11 | *Ccl21a* | chemokine (C-C motif) ligand 21a |
| B12 | *Il1f8* | interleukin 1 family, member 8 | F12 | *Ccl22* | chemokine (C-C motif) ligand 22 |
| C1 | *Il17b* | interleukin 17B | G1 | *Ccl24* | chemokine (C-C motif) ligand 24 |
| C2 | *Il17c* | interleukin 17C | G2 | *Ccl25* | chemokine (C-C motif) ligand 25 |
| C3 | *Il17d* | interleukin 17D | G3 | *Ccl27* | chemokine (C-C motif) ligand 27 |
| C4 | *Il17f* | interleukin 17F | G4 | *Ccl28* | chemokine (C-C motif) ligand 28 |
| C5 | *Bmp2* | bone morphogenetic protein 2 | G5 | *Cxcl1* | chemokine (C-X-C motif) ligand 1 |
| C6 | *Bmp4* | bone morphogenetic protein 4 | G6 | *Cxcl2* | chemokine (C-X-C motif) ligand 2 |
| C7 | *Bmp5* | bone morphogenetic protein 5 | G7 | *Cxcl3* | chemokine (C-X-C motif) ligand 3 |
| C8 | *Bmp6* | bone morphogenetic protein 6 | G8 | *Cxcl5* | chemokine (C-X-C motif) ligand 5 |
| C9 | *Bmp7* | bone morphogenetic protein 7 | G9 | *Cxcl9* | chemokine (C-X-C motif) ligand 9 |
| C10 | *Bmp8a* | bone morphogenetic protein 8A | G10 | *Cxcl10* | chemokine (C-X-C motif) ligand 10 |
| C11 | *Bmp8b* | bone morphogenetic protein 8B | G11 | *Cxcl11* | chemokine (C-X-C motif) ligand 11 |
| C12 | *Gdf5* | growth differentiation factor 5 | G12 | *Cxcl12* | chemokine (C-X-C motif) ligand 12 |
| D1 | *Gdf7* | growth differentiation factor 7 | H1 | *Cxcl13* | chemokine (C-X-C motif) ligand 13 |
| D2 | *Gdf1* | growth differentiation factor 1 | H2 | *Cxcl14* | chemokine (C-X-C motif) ligand 14 |
| D3 | *Gdf3* | growth differentiation factor 3 | H3 | *Cxcl16* | chemokine (C-X-C motif) ligand 16 |
| D4 | *Bmp3* | bone morphogenetic protein 3 | H4 | *Ppbp* | pro-platelet basic protein |
| D5 | *Gdf10* | growth differentiation factor 10 | H5 | *Actb* | Actin, beta |
| D6 | *Nodal* | nodal | H6 | *B2m* | Beta-2 microglobulin |
| D7 | *Mstn* | myostatin | H7 | *Gapd* | glyceraldehyde-3-phosphate dehydrogenase |
| D8 | *Gdf11* | growth differentiation factor 11 | H8 | *Gusb* | Glucuronidase, beta |
| D9 | *Gdf9* | growth differentiation factor 9 | H9 | *Hprt1* | Hypoxanthine guanine phosphoribosyl transferase 1 |
| D10 | *Bmp15* | bone morphogenetic protein 15 | H10 | *Pgk1* | Phosphoglycerate kinase 1 |
| D11 | *Inhba* | inhibin beta-A | H11 | *Ppia* | Peptidylprolyl isomerase A |
| D12 | *Inhbb* | inhibin beta-B | H12 | *Rpl13a* | Ribosomal protein L13a |
